# Brd4 activates P-TEFb for RNA polymerase II CTD phosphorylation

**DOI:** 10.1093/nar/gku449

**Published:** 2014-05-23

**Authors:** Friederike Itzen, Ann Katrin Greifenberg, Christian A. Bösken, Matthias Geyer

**Affiliations:** 1 Max Planck Institute of Molecular Physiology, Department of Physical Biochemistry, 44227 Dortmund, Germany; 2 Center of Advanced European Studies and Research (caesar), Group Physical Biochemistry, 53175 Bonn, Germany

## Abstract

The bromodomain protein Brd4 regulates the transcription of signal-inducible genes. This is achieved by recruiting the positive transcription elongation factor P-TEFb to promoters by its P-TEFb interaction domain (PID). Here we show that Brd4 stimulates the kinase activity of P-TEFb for phosphorylation of the C-terminal domain (CTD) of RNA polymerase II over basal levels. The CTD phosphorylation saturation levels, the preferences for pre-phosphorylated substrates, and the phosphorylation specificity for Ser5 of the CTD however remain unchanged. Inhibition of P-TEFb by Hexim1 is relieved by Brd4, although no mutual displacement with the Cyclin T-binding domain of Hexim1 was observed. Brd4 PID shows a surprising sequence motif similarity to the *trans*-activating Tat protein from HIV-1, which includes a core RxL motif, a polybasic cluster known as arginine-rich motif, and a C-terminal leucine motif. Mutation of these motifs to alanine significantly diminished the stimulatory effect of Brd4 and fully abrogated its activation potential in presence of Hexim1. Yet the protein was not found to bind Cyclin T1 as Tat, but only P-TEFb with a dissociation constant of 0.5 μM. Our data suggest a model where Brd4 acts on the kinase subunit of P-TEFb to relieve inhibition and stimulate substrate recognition.

## INTRODUCTION

Regulating the processivity of RNA polymerase II (RNAPII) mediated transcription is a key mechanism for controlling the expression of vast arrays of signal-inducible genes in metazoan (1). Shortly after transcription initiation, RNAPII pauses at the promoter-proximal region ∼50 nt downstream of the transcription start site. The cyclin-dependent kinase Cdk9 and its corresponding cyclin T (CycT) promote the transition from pausing to transcriptional elongation by phosphorylating negative factors and the C-terminal domain (CTD) of RNAPII (2). Cdk9 and CycT together constitute the positive transcription elongation factor b (P-TEFb), which phosphorylates serine residues in the repetitive hepta-repeat structure Y_1_S_2_P_3_T_4_S_5_P_6_S_7_ of the CTD (3). To control transcriptional elongation, the activity of P-TEFb is tightly regulated with the majority of P-TEFb being sequestered in an inactive 7SK small nuclear ribonucleoprotein (snRNP) complex. This multimeric complex contains 7SK snRNA, the RNA scaffolding protein Larp7, the coupling factors Hexim1 or Hexim2, and possibly the RNA 5′ capping protein MePCE (4–6). Transition from the inactive state into the catalytically active form of P-TEFb is associated with the bromodomain-containing protein 4 (Brd4) (7–9). A C-terminal segment of 54 residues in Brd4, termed P-TEFb interacting domain (PID), was identified to mediate the activation of P-TEFb from the inactive ribonucleoprotein complex (9–11). But also protein complexes including mixed lineage leukemia fusion partners or the Paf1 elongation factor were shown to activate P-TEFb (12,13). The SR-splicing factor SRSF2 was in addition recently reported to be part of the 7SK complex assembled at gene promoters and plays a direct role in transcription pause release (14).

The bromodomain and extra-terminal (BET) domain family (Brd2, Brd3, Brd4 and BrdT) shares a common domain architecture featuring two amino-terminal bromodomains that exhibit high levels of sequence conservation, and a more divergent carboxy-terminal recruitment domain (15). The bromodomain is an acetyl-lysine-binding unit, and Brd4 has been shown to associate with acetylated histones H3 and H4 and with acetylated lysines in CycT1 (16–18). Brd4 was originally identified as a ubiquitously expressed chromatin adapter that maintains epigenetic memory and regulates cell cycle progression by its association with chromosomes during mitosis (19). BET family proteins have been shown to be important players in human disease, including cancer, leukemia and viral infections. Brd4 is ascribed a key determinant in acute myeloid leukemia, multiple myeloma, Burkitt's lymphoma, NUT midline carcinoma, colon cancer, and inflammatory disease (20–25). The identification of two anticancer compounds, JQ1 and I-BET, that target the first bromodomain of Brd4, showed the potency of blocking the chromatin binding affinity of a specific BET family protein for therapeutic intervention in cancer research and inflammation (21,25–27). Besides the transcriptional effects, recent data implicate a C-terminally truncated isoform of Brd4 as an insulator of chromatin that can modulate the signaling response to DNA damage (28).

In addition, several viruses target BET proteins to regulate viral and cellular transcription. The papillomavirus E2 proteins bind to the C-terminal region of Brd4, using this interaction in tethering the viral genomes to mitotic chromosomes (29,30) and activating transcription (31,32). Retroviruses HIV and HTLV instead contain the Tat/TAR ribonucleoprotein (RNP) transactivation complex that directly interacts with CycT1 to recruit P-TEFb to RNAPII for the stimulation of viral transcripts (33). BET bromodomain inhibition turned out to reactivate HIV from latency in cell lines and primary T-cell models, suggesting a possibility to activate viral reservoirs for their eradication (34–38). Here, we show that the PID in Brd4 stimulates P-TEFb activity over basal levels even in presence of the cellular inhibitor Hexim1. This function requires the assembly of sequence motifs in PID that is reminiscent to known Tat domains. The substrate preferences for the consensus CTD or a Ser7 pre-phosphorylated CTD however remained unchanged, and also the phosphorylation specificity of Cdk9 is unaltered by Brd4 PID. The mechanism of P-TEFb activation instead appears different from HIV-1 Tat, as PID does not interact with Cyclin T1. These results identify the PID of Brd4 as a co-factor of active P-TEFb that stimulates the kinase activity but does not change its preferences.

## MATERIALS AND METHODS

### Plasmid preparations

Murine Brd4 bromodomain constructs were cloned and expressed as hexa-histidine tagged recombinant proteins in *Escherichia coli* BL21(DE3) Rosetta cells using the pProEx-HTb vector system similarly as described (17). Brd4 PID constructs were cloned from a synthetic plasmid codon optimized for expression in *E. coli* cells and ligated with *Nco*I and *Eco*RI restriction sites at the 5′ and 3′ ends, respectively, into a pGEX-4T1 TEV vector for the expression as N-terminal Glutathion S-transferase (GST)-fusion proteins. The putative kinase domain of human Brd4 1-698 was similarly cloned from a codon optimized synthesized plasmid (GeneArt) with *Nco*I/*EcoR*I restriction sites and ligated into the pGEX-4T1 TEV expression vector. In addition, the Brd4 1-698 coding gene was ligated with *EcoR*I/*Not*I restriction sites into the pACE Bac1 expression vector (ATG:biosynthetics) equipped with an N-terminal GST-fusion protein coupled with a TEV protease cleavage site. Tat expression plasmids were cloned into the pGEX-4T1 TEV expression vector containing an N-terminal GST tag for affinity purification as reported previously (39). All expression plasmids were confirmed by DNA sequencing prior to expression. Full length His-tagged Cdk9 was cloned into the pACE Bac1 vector as described (40).

### Protein expression and purification

All proteins were expressed and purified in a two-step purification strategy using affinity and size exclusion chromatography. The affinity tags were removed by TEV protease cleavage overnight before size exclusion chromatography. GST-CTD was transformed into BL21(DE3) Rosetta *E. coli* cells and expressed in TB medium by autoinduction with 0.2% lactose at 25°C overnight. After initial affinity purification via the GST tag, impurities were removed by size exclusion chromatography. Identity and homogeneity of used proteins were validated by electrospray ionization mass spectrometry (ESI-MS) and sodium dodecyl sulphate-polyacrylamide gel electrophoresis (SDS-PAGE).

P-TEFb was reconstituted from baculo virus-infected *Sf21*-expression of His-Cdk9 and *E. coli*-expressed CycT1 1-272. About 4 l of *Sf21* cells were infected with the obtained virus stock in a 1:50 ratio and incubated at 27°C for expression for 3–4 days. For purification of P-TEFb protein complex, *Sf21* cells from Cdk9 expression were lysed by sonification in buffer A (50 mM Hepes, pH 7.6, 500 mM NaCl, 10% glycerol, 5 mM β-mercaptoethanol) and cell remnants pelleted for 90 min at 45 000 × g. Purified CycT1 1-272 protein from *E. coli* expression was added in excess before and after cell lysis to form the P-TEFb complex. The protein-containing supernatant was loaded on a Ni^2+^ affinity column (GE Healthcare) and washed with buffer A with 20 mM imidazole added. The obtained complex was eluted by a linear gradient from 20 to 400 mM imidazole and analyzed by SDS-PAGE. Protein-containing fractions were concentrated and further purified by size exclusion chromatography in buffer B (50 mM Hepes, pH 7.6, 500 mM NaCl, 10% glycerol, 1 mM TCEP).

### Kinase activity assays

The kinase activity of P-TEFb was analyzed either by quantitative measurements using radioactive-labeled ATP in filter binding assays or by ESI-MS as semi-quantitative means to identify maximal phosphorylation numbers (40). For comparative analysis of kinase activity, 0.2 μM P-TEFb was incubated in kinase buffer (150 mM Hepes, pH 7.6, 34 mM KCl, 7 mM MgCl_2_, 2.5 mM DTE, 5 mM β-glycerophosphate) with 100 μM GST-substrate in 35 μl reaction volumes for 15 min. For time course saturation experiments, only 10 μM GST-substrate was used. For analysis of P-TEFb activation, Brd4 proteins were added at a concentration of 10 μM to the reactions. In case of P-TEFb inhibition by Hexim1, 2 μM Hxm1 200-359 or flavopiridol was added prior to the reaction start. Kinase reactions were started by addition of 2 mM ATP, incubated at 30°C and 300 rpm and stopped by addition of ethylenediaminetetraacetic acid (EDTA). In each experimental series, the same amount of CTD substrates, either as GST-CTD or as pre-phosphorylated CTD peptides, was used for direct comparison of kinase activities.

### Analysis of CTD phosphorylation

For ESI-MS detection of quantitative phosphorylation numbers, kinase reactions were performed with only 50 mM Hepes buffer, as reduction of buffer reagent led to improved signal quality. The kinase reaction was stopped by adding excess of EDTA or flavopiridol before the solution was subjected to ESI-MS analysis. Alternatively, reactions were qualitatively analyzed by SDS-PAGE for changed migration behaviors caused by phosphate incorporation. Quantitative measurements of the kinase catalytic activity were carried out in the presence of [^32^P]-labeled γ-ATP (Perkin Elmer) and stopped by spotting 15 μl of the reaction to P81 phospho-cellulose paper (OmniLab). Surplus reaction buffer and ATP were removed by three washing steps in 0.75% phosphoric acid and sample radioactivity was determined as a measure for incorporated [^32^P]-phosphate.

### Western blot analysis

For Western blot analysis, kinase assays were run with 0.2 μM P-TEFb, 10 μM Brd4 PID, 10 μM GST-CTD_[52]_ and 2 mM ATP in kinase buffer. After incubation with P-TEFb or P-TEFb–Brd4 PID, GST-CTD proteins were subjected to SDS-PAGE on a 12% gel before transfer to nitrocellulose (GE Healthcare). Monoclonal antibodies specific for RNAPII CTD phosphorylations pSer2 (3E10), pSer5 (3E8) or pSer7 (4E12) were used as described previously (40). Membranes were stained with chicken anti-rat IgG HRP secondary antibodies and binding revealed by enhanced chemiluminescence.

### Protein binding experiments

Isothermal titration calorimetry (ITC) measurements were carried out using an iTC_200_ calorimeter (GE Healthcare). Brd4 PID and P-TEFb proteins were dialyzed overnight at 4°C against binding buffer (50 mM Hepes, pH 7.6, 250 mM NaCl, 5% glycerol, 1 mM MgCl_2_, 1 mM TCEP) to ensure equal buffer contents. Brd4 PID at a concentration of 100 μM was stepwise titrated into 10 μM of either P-TEFb complex or CycT1 1-272 protein and changes in cell temperature were used to calculate the thermodynamic parameters of a potential binding using the software provided by the manufacturer.

## RESULTS

### Brd4 PID stimulates P-TEFb kinase activity over basal levels

**Figure 1.**
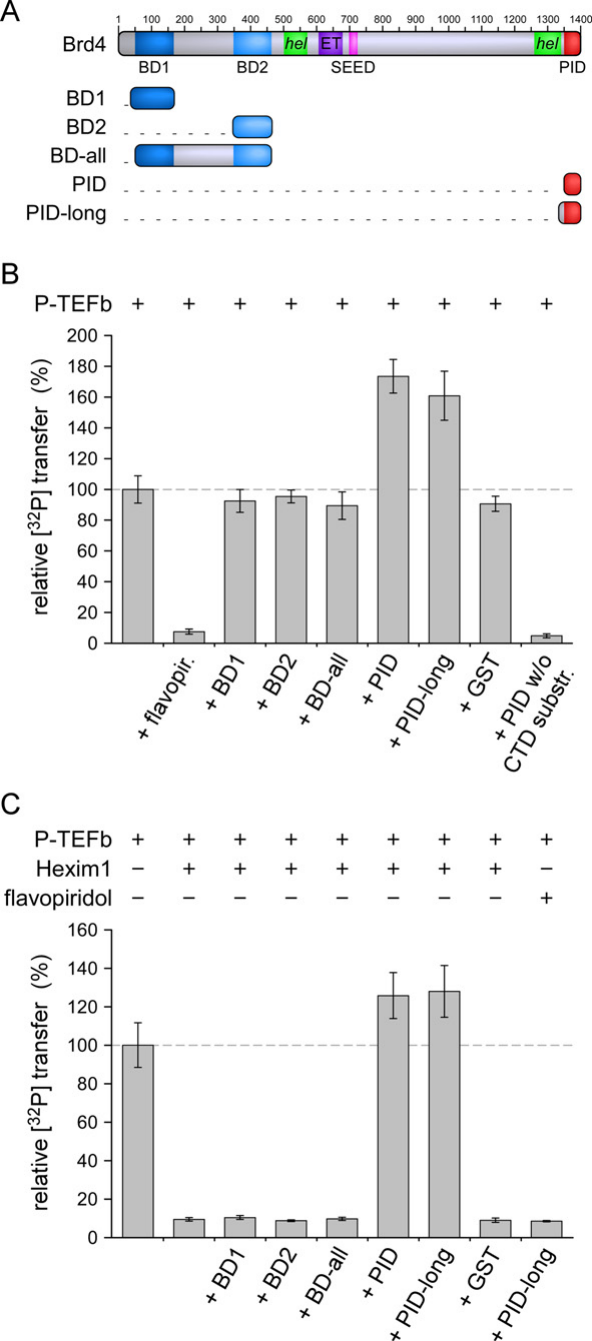
Brd4 PID is an activator of basal and Hexim1-inhibited P-TEFb. (**A**) Modular domain architecture of murine Brd4. Five different protein constructs were generated that contained the N-terminal bromodomains BD1 and BD2 or the C-terminal P-TEFb interaction domain (PID). Human Brd4 has a total length of 1362 amino acids, whereas mouse Brd4 contains 1400 residues, primarily resulting from an insert of 33 residues at position 1098. Domains are annotated below the diagram with a helical region indicated. (**B**) Stimulation of P-TEFb activity for RNAPII CTD phosphorylation by Brd4 proteins. The two PID fragments but not the bromodomain containing fragments of Brd4 enhanced the catalytic activity of P-TEFb. PID itself however is not a substrate of P-TEFb. (**C**) The stimulating effect of PID is even more pronounced upon inhibition of P-TEFb by Hexim1 200-359. Only the PID containing constructs were able to relieve the inhibited state, but not the bromodomains or GST as a control. In contrast, Brd4 PID was unable to activate P-TEFb that has been inhibited with flavopiridol, suggesting a different mechanism of inhibition for the ATP-competitive compound flavopiridol and the cellular regulation factor Hexim1.

Five different expression constructs of Brd4 proteins were generated and tested for their efficacy of P-TEFb activation. These constructs contained the N- and C-terminal bromodomains of Brd4, designated as BD1 and BD2, a combined bromodomain construct of 45 kD size encompassing both bromodomains including the intermediate linker sequence, designated as BD-all, and two variants of the C-terminal sequence motif encompassing either 50 or 67 residues, designated as PID and PID-long, respectively. The latter construct was N-terminally elongated for better purification strategies and more accurate determination of the protein concentration. The domain annotations and construct boundaries are shown in Figure 1A. All Brd4 proteins were expressed in *E. coli* and purified to homogeneity.

The effect of Brd4 proteins on P-TEFb activity was tested in the presence or absence of Hexim1. First, a kinase activity assay was performed using radioactive-labeled ATP as co-substrate and a GST-tagged RNAPII CTD protein consisting of nine hepta-repeats as substrate. The kinase reaction was incubated for 15 min before being washed and spotted onto filter paper for measurement of the radioactive content. Using 50-fold excess of Brd4 proteins over P-TEFb at 0.2 μM concentration, no change in activity was observed for either BD1, BD2 or the combined BD-all construct. In contrast, the C-terminal constructs PID and PID-long both stimulated P-TEFb activity up to 1.74- and 1.61-fold over basal levels, respectively (Figure 1B). Addition of GST in similar concentrations to Brd4 proteins in a control experiment did not affect the kinase reaction. As a second control, PID was tested for its capability as potential P-TEFb substrate. Omitting the CTD substrate in this experiment, no contributing effect of the Brd4 protein alone to the total radioactive counts in the kinase activity assay was seen. These results established Brd4 PID as a co-factor of P-TEFb that helped stimulating CTD phosphorylations.

A similar set of experiments was performed in the presence of the cellular P-TEFb inhibition factor Hexim1. As before, P-TEFb was used at 0.2 μM concentrations whereas an N-terminally truncated form of Hexim1 (Hxm1 200-359) that showed a similar inhibitory potential as the full length Hexim1/7SK snRNA complex (40) was added at 2 μM concentration to P-TEFb prior to incubation with Brd4 proteins at 10 μM concentration. As observed before, Hexim1 potently reduced the kinase activity of P-TEFb to less than 10% (Figure 1C). Notably, this inhibition could be overcome by addition of Brd4 PID in both variants, underlining the P-TEFb activating potential of the bromodomain-containing protein. Again, this effect was only seen for the Brd4 C-terminal constructs, but not for the bromodomain constructs BD1, BD2 or BD-all (Figure 1C). In presence of Hexim1, PID raised the P-TEFb activity up to 1.3-fold over basal levels confirming its stimulatory effect for CTD substrate modifications. In contrast, when P-TEFb was inhibited with the ATP competitive compound flavopiridol at 2 μM concentrations, Brd4 PID was unable to relieve the inhibition as well as all other Brd4 constructs tested (Figure 1C and Supplementary Figure S1). This indicates that the mechanism of inhibition is different for the compound flavopiridol compared to the protein Hexim1 and that Brd4 can act only on the cellular regulator but not the inhibiting compound.

### Kinetics of the Brd4 PID stimulatory effect on P-TEFb

**Figure 2.**
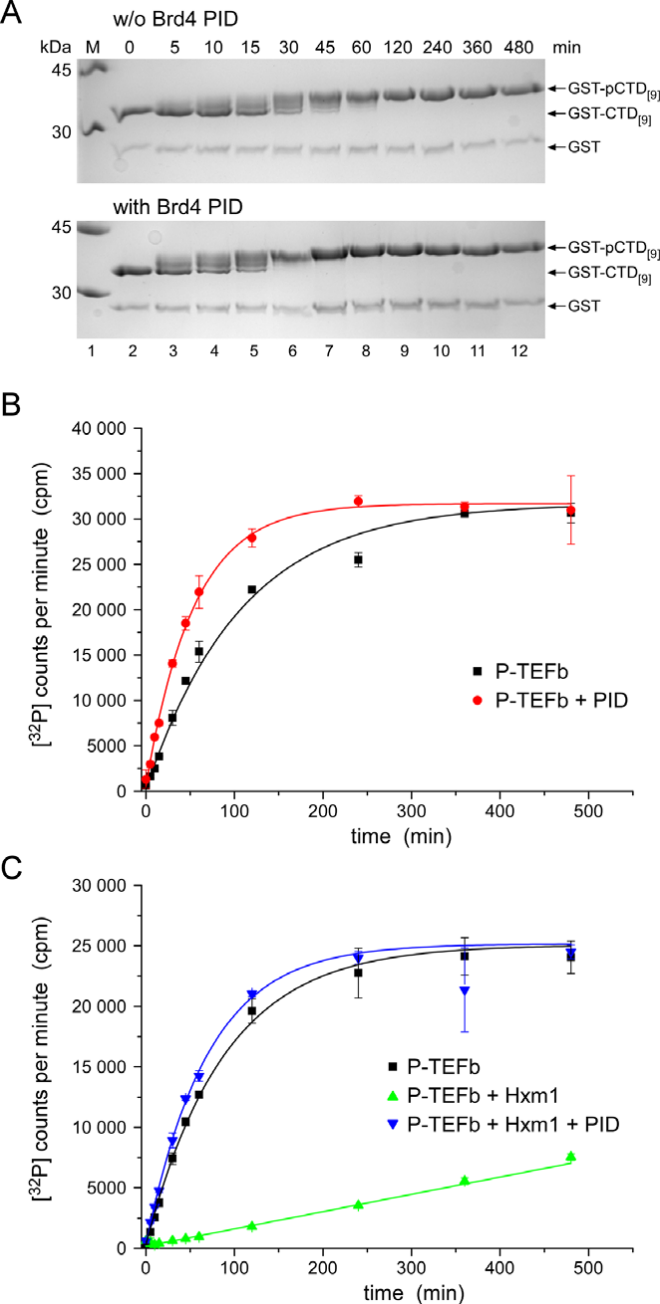
Kinetics of Brd4 PID stimulated P-TEFb activity. (**A**) SDS-PAGE analysis of P-TEFb-mediated GST-CTD phosphorylation in the absence (upper panel) and presence (lower panel) of PID. The phosphorylation reaction is significantly enhanced in the presence of the PID. (**B**) Time course experiments revealed a 2-fold higher catalytic efficiency for the P-TEFb-PID complex compared to P-TEFb alone. (**C**) Inhibition of P-TEFb by Hexim1 is revived by the presence of PID. Even here, the gain in catalytic efficiency is still over basal levels of P-TEFb alone.

The stimulatory effect of P-TEFb-mediated CTD phosphorylation in presence of Brd4 PID could result either from changed substrate specificity that might give access to more phosphorylation sites on the CTD substrate or from an increased enzymatic activity of the P-TEFb–Brd4 PID complex for the substrate. To challenge these hypotheses, we performed time resolved kinase activity experiments by SDS-PAGE analysis and quantitative radioactivity measurements. The stimulatory effect of Brd4 PID on P-TEFb is already seen in the migration profiles of phosphorylated GST-CTD substrates resolved in a time course experiment (Figure 2A). Quantitative experiments were performed at 0.2 μM enzyme concentration using a CTD substrate of nine hepta-repeats at 10 μM concentration and excess of 1 mM ATP. In the absence of Hexim1, P-TEFb readily phosphorylated the CTD substrate with a *K*_cat_/*K*_M_ value of 0.77 × 10^3^ M^−1^ s^−1^, or 6.9 × 10^3^ M^−1^ s^−1^ when multiplied for the number of nine phosphorylation sites present in the substrate template (Figure 2B). Addition of Brd4 PID to the reaction led to increased counts in the beginning of the reaction with a *K*_cat_/*K*_M_ value of 1.55 × 10^3^ M^−1^ s^−1^ (or 1.4 × 10^4^ M^−1^ s^−1^ corrected for the nine sites). The amplitude upon saturation instead remained similar indicating that the number of phosphorylations in the CTD substrate was not increased (Figure 2B). Therefore, PID increased the catalytic activity of P-TEFb for CTD phosphorylation by 2-fold, but did not led to a higher number of kinase accessible phosphorylation sites in the substrate in general.

In the presence of Hexim1, the stimulatory effect of Brd4 PID to overcome P-TEFb inhibition is similarly visible with *K*_cat_/*K*_M_ values of 0.94 × 10^3^ M^−1^ s^−1^ for the basal activity of P-TEFb and 1.20 × 10^3^ M^−1^ s^−1^ for the tripartite assembly of P-TEFb, Hexim1 and Brd4 PID (Figure 2C). Again, the amplitudes for the CTD modifying reactions upon saturation were similar, indicating equitable phosphorylation reactions on the substrate template. This result was confirmed by mass spectrometry analyses, which showed an equal number of phosphorylations for basal P-TEFb activity and P-TEFb–Brd4 PID stimulated activity (Supplementary Figure S2).

### Brd4 PID exhibits a sequence motif composition similar to viral Tat proteins

**Figure 3.**
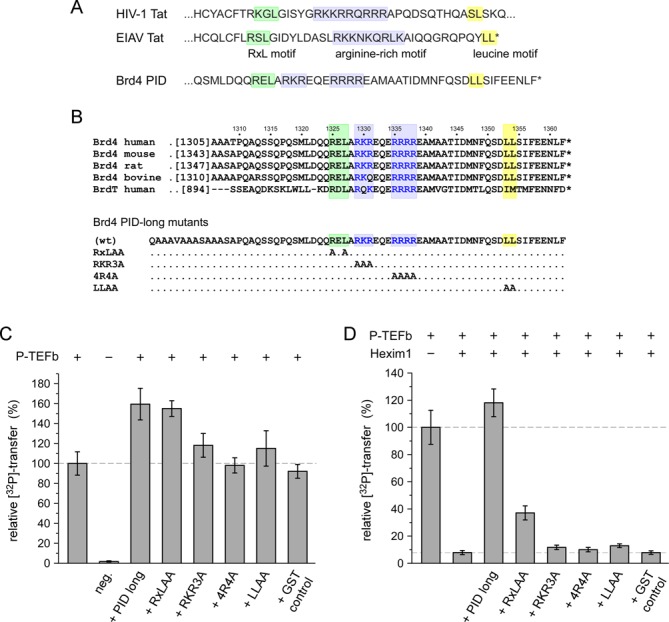
Brd4 PID shares sequence motif similarity to viral Tat proteins. (**A**) The Brd4 PID contains a surprising similarity in sequence motif assembly to viral Tat proteins, including an RxL motif, a polybasic cluster and a C-terminal leucine motif. (**B**) Alignment of Brd4 PID sequences from different organisms and generation of four PID mutants. (**C**) Mutations of the characteristic sequence motifs to alanine impair PID function for P-TEFb stimulation. The RxLAA mutant showed only a small decrease in stimulatory function, whereas mutation of the four arginines (4R4A) fully abrogated the PID stimulatory effect. (**D**) Upon inhibition by Hexim1 (200-359), the contribution of the PID sequence motifs emerges showing that the polybasic cluster and the leucine motif of PID are necessarily required for P-TEFb activation by Brd4.

Besides Brd4, the Tat protein from HIV and SIV retroviruses is known to activate P-TEFb. Together with its corresponding transcription activation response element TAR, the *trans*-activating Tat protein recruits P-TEFb to RNAPII for the activation of transcriptional elongation of viral genes (41). When analyzing Brd4 PID sequences we noticed surprising similarities in the composition of sequence motifs with viral Tat proteins. This includes a central core sequence with an RxL motif, which is known from other Cdk/Cyclin pair interacting regulators and substrates as p27^KIP^ and Cdc6 (42), a basic cluster known as arginine-rich motif (ARM) in Tat for TAR binding, and a short hydrophobic sequence motif about 10 residues downstream of the basic cluster (Figure 3A). Structural analyses from equine infections anemia virus (EIAV) showed that the two flanking motifs to the central basic residues interact with the first cyclin box repeat of CycT1, while the central ARM is free to interact with the major groove of the double-strand stem loop regions of the RNA (43). In the following experiment series, the four prominent sequence motifs RxL, RKR, RRRR and LL were mutated to alanine, termed RxLAA, RKR3A, 4R4A and LLAA, respectively, to probe their effects on the Brd4 PID-mediated P-TEFb activation (Figure 3B).

As before, the capability of PID for P-TEFb stimulation was tested in the presence and absence of Hexim1 as the negatively regulating factor. Whereas wild type PID stimulated P-TEFb activity to similar extent (160%) as before, mutations RKR3A, 4R4A and LLAA decreased the hyper-activation effects to 120%, 100% and 118%, respectively, in the absence of Hexim1 (Figure 3C). The smallest effect was seen for the RxLAA mutant that barely impaired PID-mediated hyper-activation. The effects of Brd4 PID mutations became however much more accentuated in the presence of Hexim1. Now, PID mutants RKR3A, 4R4A and LLAA almost fully lost their capability of recovering P-TEFb activity in the presence of the inhibitor, while only RxLAA showed some residual activity toward P-TEFb activation (Figure 3D). The presence of the two basic clusters and the C-terminal hydrophobic motif is therefore necessary for the functioning of Brd4 PID as P-TEFb activator; yet it does not explain if PID acts mechanistically similar on P-TEFb as the viral Tat protein.

### Conserved Tat motifs are required for P-TEFb activation

**Figure 4.**
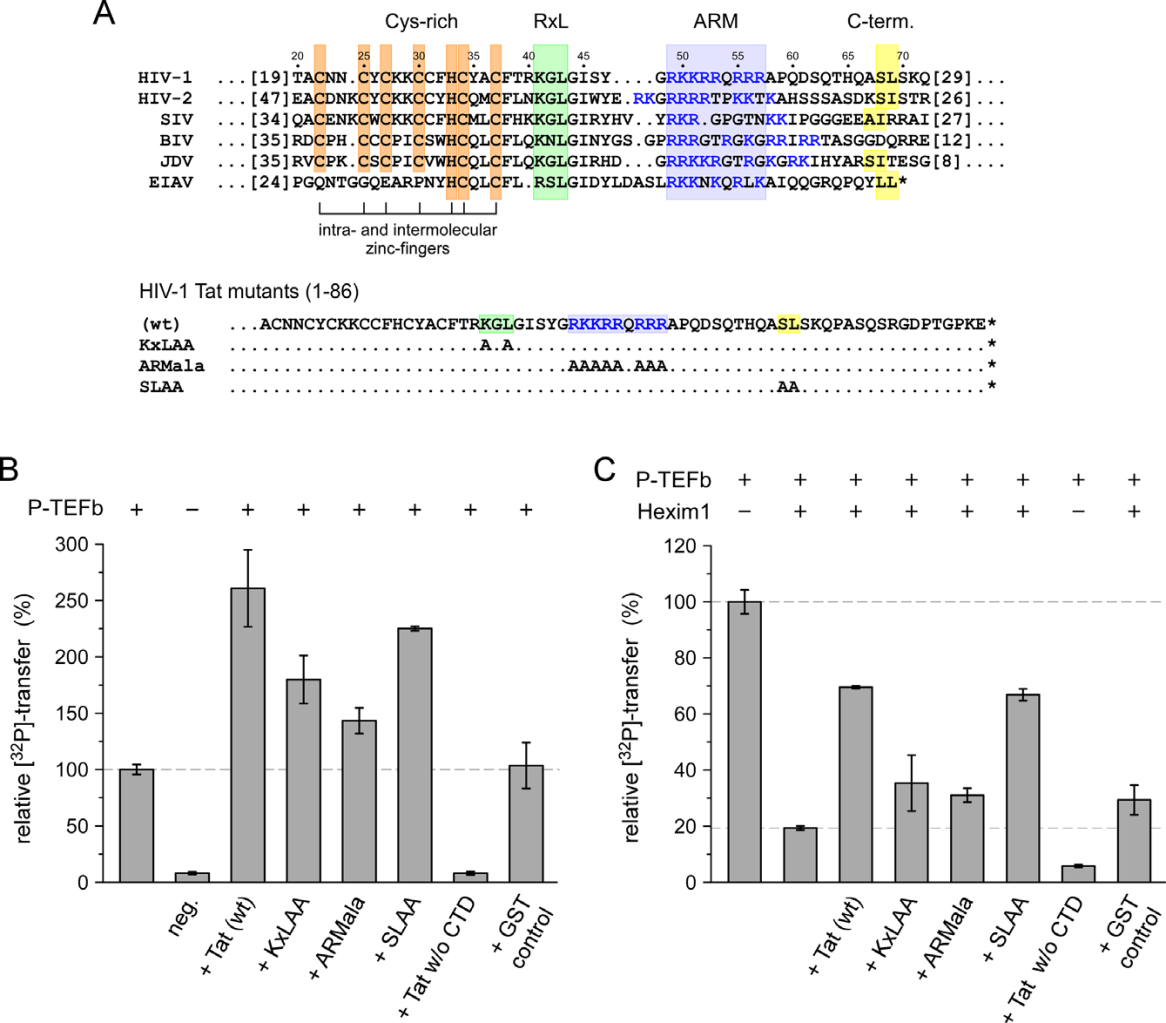
Analysis of Tat sequence motifs for P-TEFb activation. (**A**) Sequence alignment of Tat proteins from viruses HIV-1, HIV-2, SIV, BIV, JDV and EIAV. A cysteine-rich region, an RxL motif, an arginine-rich motif and a C-terminal leucine motif are conserved in viral Tat proteins. Besides wild type HIV-1 Tat (1-86), three mutants were generated and analyzed for P-TEFb activation. (**B**) Addition of HIV-1 Tat potently stimulated P-TEFb activity for CTD phosphorylation up to 2.5-fold. Mutation of the KxL motif and the ARM, but to a lesser extent the SL motif, diminished the stimulatory effect of Tat. Yet Tat is not a substrate of P-TEFb as shown by a reaction missing the RNAPII CTD. (**C**) In the presence of Hexim1, Tat is still able to activate P-TEFb but not as potently as in the absence of the cellular regulator. The KxL motif and the ARM are necessarily required for this activating function, but not the SL motif.

A sequence alignment of viral Tat proteins from HIV-1, HIV-2, SIV, BIV, JDV and EIAV shows that a cysteine-rich region, a central region containing a KxL motif, the ARM, and a C-terminal leucine motif are conserved in viral Tat proteins (Figure 4A). To compare the effects of sequence motifs between Brd4 and Tat, we mutated the conserved motifs KxL, ARM and SL in HIV-1 Tat (1-86) to alanine, termed KxLAA, ARMala and SLAA (Figure 4A). Of note, the importance of the cysteine-rich region for the capability of Tat for transactivation was shown in multiple studies before (44). Addition of wild type HIV-1 Tat in the *in vitro* kinase assay potently stimulated P-TEFb activity for CTD phosphorylation up to 2.5-fold (Figure 4B). Mutation of the KxL motif and the ARM diminished the stimulatory effect of Tat, whereas the SLAA mutation showed only a small effect in diminishing the stimulatory effect of Tat. Tat itself is not a substrate of P-TEFb as shown by a reaction missing the RNAPII CTD. Again, the importance of the conserved motifs for Tat functioning became apparent upon P-TEFb inhibition by Hexim1 200-359. In contrast to Brd4 PID, which still had a stimulatory effect, wild type Tat recovered P-TEFb activity only to 70% in the presence of Hexim1 (Figure 4C). This ability was lost for the KxLAA and the ARMala mutants, while the SLAA mutant fully maintained the activating phenotype. To conclude, the RxL or KxL motif and the polybasic cluster exhibit similar importance in Brd4 PID and Tat for P-TEFb activation, whereas the LL motif in Brd4 PID is fully required for activation, but the SL motif in Tat not. These differences point to possibly different modes in the activation mechanism of Brd4 and viral Tat proteins for Hexim1-inhibited P-TEFb.

### Brd4 PID interacts with P-TEFb but not with CycT1 alone

**Figure 5.**
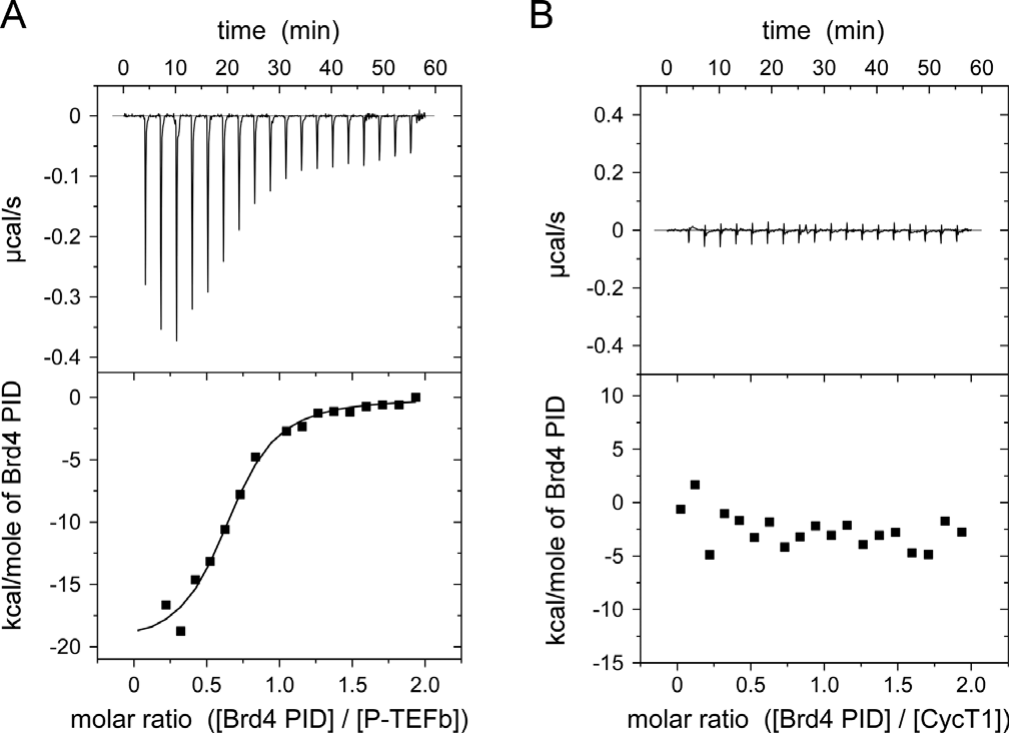
Interaction of Brd4 PID with P-TEFb. (**A**) Isothermal titration calorimetry measurements of Brd4 PID with P-TEFb (Cdk9/CycT1) revealed a dissociation constant of 0.47 μM for this interaction. (**B**) In contrast, PID showed no binding to CycT1 alone, suggesting Cdk9 as the targeting moiety for Brd4 binding. The thermodynamic parameters of the interaction are listed in Table 1.

Binding of the *trans*-activator Tat and the inhibitor Hexim1 are mutually exclusive processes on the same surface of CycT1, leading to a displacement of Hexim1 under physiological conditions due to the higher affinity of Tat for the CycT1 protein (45,46). We therefore determined binding affinities and thermodynamic parameters of Brd4 PID for interactions with CycT1 and P-TEFb by ITC. Of note, Cdk9 alone could not be tested for binding experiments without its corresponding CycT1 subunit due to its poor stability upon purification. Using concentrations of 10 μM P-TEFb bound to the non-hydrolysable ATP analog AppNHp and 100 μM PID, which was injected from a syringe to P-TEFb, we determined a dissociation constant of 470 ± 140 nM for the P-TEFb-PID interaction (Figure 5A). In contrast, no binding was observed for PID when subjected to CycT1 alone (Figure 5B). This finding differs significantly from previous results with HIV-1 Tat, which showed that Tat readily interacted with the cyclin box repeats of CycT1 and the groove between both cyclin boxes (41,43,47).

**Table 1.** Thermodynamic parameters of PID binding to P-TEFb determined by isothermal titration calorimetry

Titration scheme^a^	*K*_d_ (μM)	Δ*H* (kcal/mol)	*T*Δ*S* (kcal/mol)	Molar ratio *n*
100 μM Brd4 PID to 10 μM P-TEFb	0.47 ± 0.14	−20.1 ± 1.2	−11.48	0.65 ± 0.03
100 μM Brd4 PID to 10 μM CycT1	–	–	–	–

^a^The measurements were performed at a temperature of 298 K.

As a second independent experiment, we performed GST-pull down precipitation experiments with either Tat/TAR or Brd4 PID using GST-labeled CycT1 and the Cyclin T-binding domain (TBD) of Hexim1 in the supernatant. Similarly as observed before (46), increasing concentrations of Tat/TAR led to the disappearance of Hexim1 TBD and the appearance of Tat in the GST-CycT1 precipitations, revealed by Coomassie stained SDS-PAGE analysis (Supplementary Figure S3A). In contrast, neither binding of Brd4 PID to CycT1 nor disappearance of Hexim1 TBD was observed upon titrations with increasing amounts of Brd4 PID (Supplementary Figure S3B). From these experiments we conclude that the activation mechanism and the stimulatory effect of Brd4 PID for P-TEFb are different from Tat and the Tat/TAR complex.

### CTD substrate preferences of P-TEFb–Brd4 PID

**Figure 6.**
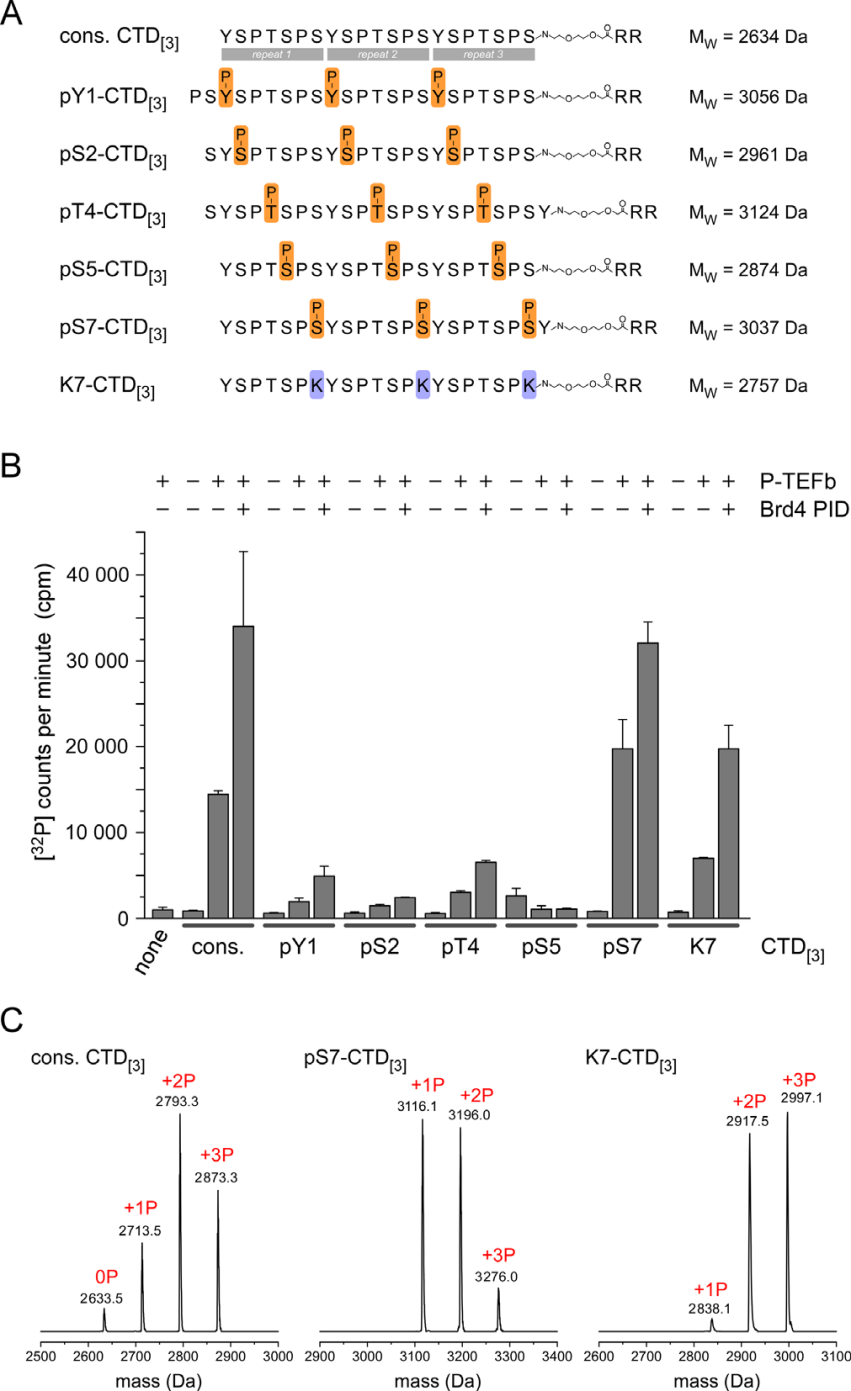
Brd4 PID increases the activity of P-TEFb for specific CTD substrates. (**A**) RNAPII CTD substrate peptides used for P-TEFb activity measurements contained three consensus hepta-repeats with either no modifications (cons. CTD_[3]_), phosphorylation marks continuously set at Tyr1 (pY1-CTD_[3]_), Ser2 (pS2-CTD_[3]_), Thr4 (pT4-CTD_[3]_), Ser5 (pS5-CTD_[3]_) or Ser7 (pS7-CTD_[3]_). In addition, a lysine residue was set at position 7 as the most common variation of the consensus CTD (K7-CTD_[3]_). (**B**) Substrate preferences of P-TEFb alone or in presence of Brd4 PID. 100 μM of CTD peptides were incubated with 0.2 μM P-TEFb, 1 mM ATP and 2 μM Brd4 PID at 30°C. The recognition of P-TEFb for the various CTD peptides remained unchanged in the P-TEFb-PID complex, but the catalytic activity is significantly enhanced. The phosphorylation efficacy was monitored after 15 min reaction time. (**C**) ESI-MS analyses of CTD peptides after 4 h incubation with P-TEFb in presence of PID. Up to three phosphorylations were detected for the three repeat containing substrates cons. CTD_[3]_, pS7-CTD_[3]_ and K7-CTD_[3]_.

The stimulatory effect of Brd4 PID for P-TEFb could affect the recognition specificity for CTD substrates as binding of PID might change its interaction surface. We therefore analyzed a library of synthesized CTD substrates that reflect different modification states of the consensus sequence YSPTSPS as well as the prevailing alteration YSPTSPK (K7-CTD_[3]_) in distal CTD repeats. Besides the consensus substrate (cons. CTD_[3]_), a template of three repeats was continuously modified by phosphorylations at either Tyr1, Ser2, Thr4, Ser5, or Ser7, respectively (Figure 6A). The ability of P-TEFb to modify these substrates was tested in the presence of Brd4 PID in a radioactive kinase assay and quantified by mass spectrometry. The results were compared to the basal activity and specificity of P-TEFb alone. Similarly as with the longer CTD substrates used before, PID stimulated the catalytic activity of P-TEFb by about 2-fold after 15 min reaction time (Figure 6B). Likewise, the peptide containing lysine at position 7 was phosphorylated by P-TEFb and the stimulatory effect was even enhanced in presence of PID with an approximate 2.4-fold increase.

Intriguingly, out of the five pre-phosphorylated peptides tested, only the pSer7 peptide showed a strong response as a P-TEFb-PID substrate. The pSer7 mark not only stimulated the recognition of the CTD as P-TEFb substrate similarly as observed before (40), but also PID was still able to enhance the catalytic activity of P-TEFb for this substrate. In contrast, the other pre-phosphorylated peptides pTyr1, pSer2, pThr4 and pSer5 were not effectively phosphorylated by P-TEFb or the P-TEFb-PID complex, suggesting that such combinatorial phosphorylation patterns of CTD substrates are not favored by the transcription elongating P-TEFb kinase. When the phosphorylation reaction of the peptide substrates was incubated sufficiently long to achieve saturation, the consensus peptide, the pSer7 and the K7 peptide all showed similar quantitative amounts of phosphorylations. The number of phosphorylations in these peptides was analyzed by ESI mass spectrometry, which revealed up to three phosphorylations for cons. CTD_[3]_, pSer7-CTD_[3]_ and K7-CTD_[3]_ each (Figure 6C). Overall, these data confirm an elaborate preference of the P-TEFb kinase for CTD substrates that is enforced but not changed by its activator Brd4 PID.

### Phosphorylation specificity of P-TEFb–Brd4 PID

**Figure 7.**
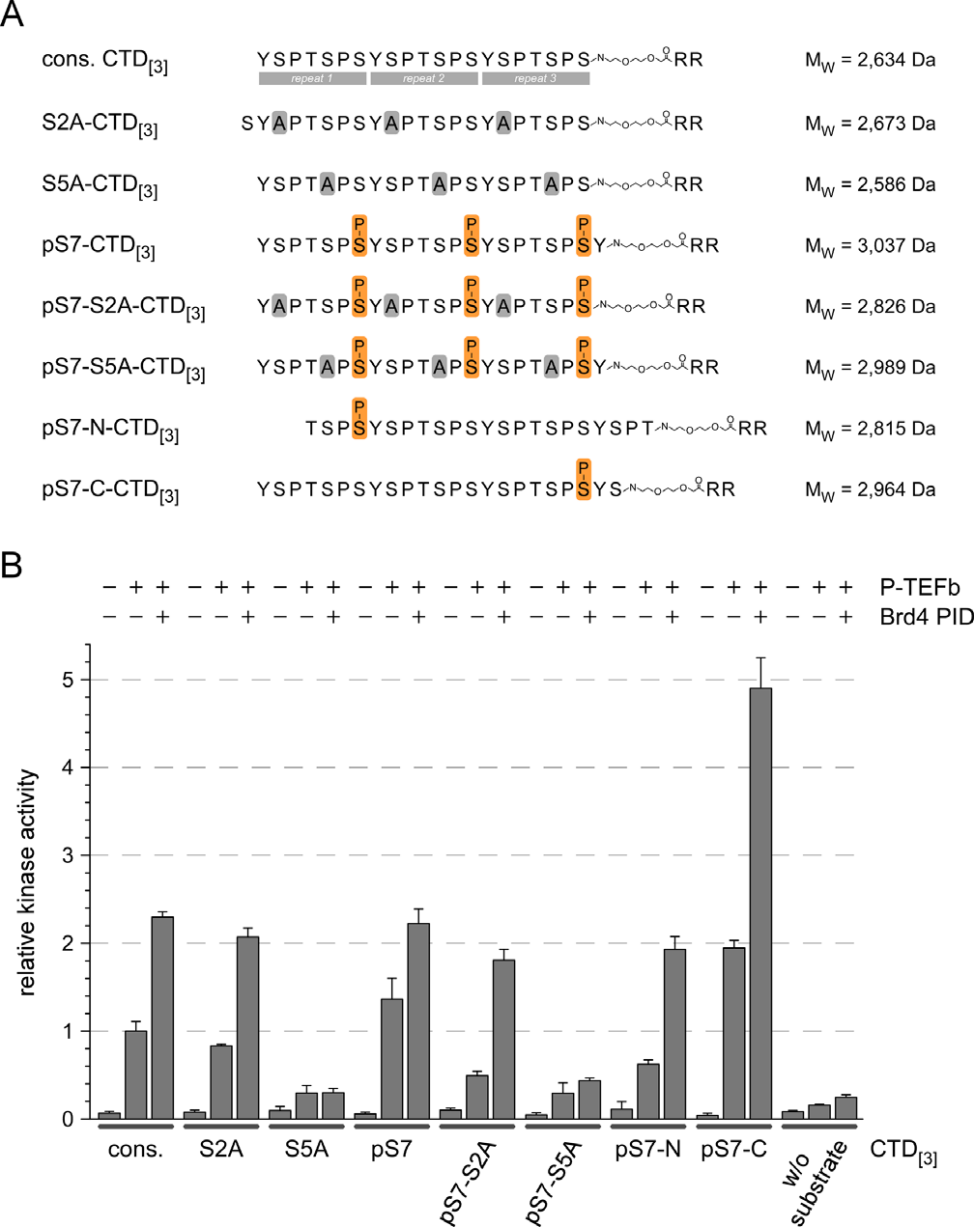
Phosphorylation specificity of P-TEFb in the absence and presence of Brd4 PID. (**A**) Using the same design of three CTD hepta-repeats as before, continuous alanine mutations were introduced at either Ser2 or Ser5 positions in the consensus CTD (cons. CTD_[3]_) or in a CTD pre-phosphorylated at position Ser7 (pS7-CTD_[3]_). In addition, a single Ser7 phosphorylation was set either in the N- or C-terminal repeat of the CTD for its recognition by P-TEFb. (**B**) Whereas the S2A variants of the CTD both in the non-phosphorylated and the Ser7 pre-phosphorylated form are readily phosphorylated by P-TEFb and the P-TEFb–Brd4 PID complex, the S5A mutants failed being recognized as P-TEFb substrates. The highest stimulatory effect with almost 5-fold increase upon addition of PID is achieved for a substrate peptide containing only one C-terminal Ser7 phosphorylation mark.

The substrate preferences described above for P-TEFb bound to Brd4 PID do not explain which residue of the hepta-repeat is in fact phosphorylated by Cdk9. Using the same peptide scaffold of three CTD repeats as before, continuous alanine mutations were placed either at Ser2 or Ser5 positions in both the consensus CTD or a CTD substrate pre-phosphorylated at position Ser7 (Figure 7A). In addition, a single Ser7 phosphorylation was set either in the N- (pS7-N-CTD_[3]_) or C-terminal repeat (pS7-C-CTD_[3]_) of the substrate peptide to test its stimulatory effect. These substrate peptides were incubated with P-TEFb or the P-TEFb–Brd4 PID complex for 15 min in the kinase assay using radioactive ATP co-substrate. Whereas the S2A variants of the CTD both in the non-phosphorylated and in the Ser7 pre-phosphorylated form got readily phosphorylated by the two kinase complexes, the two S5A peptides S5A-CTD_[3]_ and pS7-S5A-CTD_[3]_ exhibited no phosphorylation susceptibility (Figure 7B). This observation suggests that Ser5 is phosphorylated by P-TEFb and that the phosphorylation specificity is not changed by Brd4 PID. The highest stimulatory effect by Brd4 PID however was observed for a substrate peptide containing only one Ser7 phosphorylation mark in the C-terminal repeat (Figure 7B). Indeed, a 5-fold increase is achieved compared to the non-modified consensus peptide, suggesting that P-TEFb activated by Brd4 PID preferentially phosphorylates the CTD N-terminal to an existing Ser7 phosphorylation.

**Figure 8.**
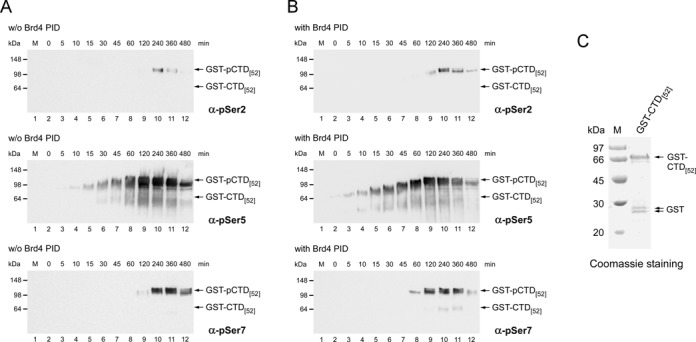
The P-TEFb–Brd4 PID complex is a Ser5 CTD kinase. (**A**) Western blot analysis of a full length GST-CTD substrate containing all 52 human repeats revealed that P-TEFb predominantly phosphorylates Ser5 of the CTD. Ser7 is phosphorylated to a lesser extent and only a faint band appeared for Ser2 phosphorylation. Shown is a time course experiment over 8 h using antibodies against pSer2 (3E10, top panel), pSer5 (3E8, middle panel) and pSer7 (4E12, lower panel). (**B**) In the presence of Brd4 PID, the phosphorylations appear earlier in the time course experiments in line with the stimulatory effect of the PID, but the phosphorylation specificity remains unchanged. Note the migration of the CTD from the hypo-phosphorylated IIa form at the beginning of the reaction (5 min) to the hyper-phosphorylated IIo form with the highest levels after 120 min. (**C**) Input control of the GST-CTD_[52]_ substrate. A coomassie stained SDS-PAGE analysis of the full length hepta-repeat region of human RNAPII CTD is shown, indicating minor fractions of leaky GST but no major hepta-repeat truncations.

To determine directly the site of phosphorylation on the CTD by P-TEFb–Brd4 PID we performed western blot analysis of a full length GST-CTD substrate containing all 52 repeats of human RNAPII using specific anti-pSer2, anti-pSer5 and anti-pSer7 antibodies, respectively. Time course experiments followed over 8 h showed that P-TEFb predominantly phosphorylates Ser5 of the CTD, while Ser7 is phosphorylated to a lesser extent and only a faint band appeared for Ser2 phosphorylation (Figure 8A). In the presence of Brd4 PID, the phosphorylations appeared earlier in line with the stimulatory effect of the PID but the phosphorylation specificity remained unchanged (Figure 8B). The stimulatory effect of Brd4 can be seen also by the shift in the migration of the CTD from the hypo-phosphorylated form (IIa) at the beginning of the reaction to the hyper-phosphorylated form (IIo), reaching the highest levels after 120 min.

A recent study described mouse Brd4 as an atypical kinase phosphorylating Ser2 of the CTD (48,49). We generated two expression constructs of the proposed kinase domain 1-698 of human Brd4 for *E. coli* bacterial cell and baculo virus infected *Sf21* insect cell expression (Supplementary Figure S4). However, no phosphorylation activity was seen for each of the two Brd4 proteins and also addition of the bromodomain inhibitor JQ1 had no effect on Brd4 (Supplementary Figure S5). Preparation of the recombinant Brd4 kinase domain might thus be more elaborate or require co-expression of activating kinases such as Cdk7 or CAK1 as shown for CTD kinases Cdk9 and Cdk12 (50,51).

## DISCUSSION

In this study we show that the PID of Brd4 comprising 50 residues not only activates P-TEFb in the presence of the cellular inhibitor Hexim1 but also enhances its catalytic activity for RNAPII CTD phosphorylation. Such ‘hyper’-activation could increase the phosphorylation reaction in the cell when Brd4 is tethered to histone structures by its N-terminal bromodomains while the C-terminal PID recruits P-TEFb to the site of transcription. The stimulatory effect of Brd4 PID is particularly high for a substrate that contains a pre-set Ser7 phosphorylation mark in a C-terminal repeat, suggesting that Brd4 activated P-TEFb preferentially extends Ser5 phosphorylations N-terminally to an existing pSer7 mark. The phosphorylation signature of P-TEFb for CTD modifications however remained unchanged upon activation by Brd4 PID. This holds true both for the preferences of substrates that are recognized by the complex as well as for the phosphorylation specificity of the Cdk9 kinase for serine residues within the CTD substrate.

P-TEFb as well as the P-TEFb-PID complex predominantly phosphorylates Ser5 of the CTD, particularly in combination with Ser7 pre-phosphorylations. This observation agrees well with the function of Brd4 activated P-TEFb for the transition from paused polymerases to transcriptional elongation, whereas Ser2 phosphorylation is associated with splicing and RNA 3′ end processing (52). The number of phosphorylations upon saturation of the reaction did not change for P-TEFb or the P-TEFb-PID complex, which shows that Brd4-mediated transcription stimulation is not achieved by phosphorylating more serine residues of the CTD. The mechanism of P-TEFb activation by Brd4 yet seems to be different from those of the HIV Tat protein, which was shown to displace the inhibiting cellular factors Hexim1 and 7SK by mutually exclusive binding to the same surface on CycT1 (45,46,10). Instead we find that Brd4 PID does not interact with the cyclin box domain of CycT1 but only with the Cdk9/CycT1 complex suggesting a direct interaction with the kinase domain.

A two-pronged binding mechanism has been proposed between Brd4 and P-TEFb, including the bromodomains and the PID of Brd4 for simultaneous binding to P-TEFb (11). This interaction is mediated upon acetylation of four lysine residues in the central region of CycT1 following the cyclin box domain (53). The second bromodomain of Brd4 was indeed found to bind tri-acetylated CycT1 (K380ac, K386ac, K390ac) with binding affinities equaling the recognition of acetylated histone tails (53,54). As the PID is likely to bind Cdk9 based on the ITC measurements performed here, the bromodomains and the PID of Brd4 may interact synergistically with both subunits of P-TEFb, providing a stable clamp of two interaction domains that are almost 900 amino acids apart. It should be noted that in the binding assays performed in this study, only recombinant protein expressions of the cyclin box domain of CycT1 were used, lacking post-translational modifications such as lysine acetylation. The acetylation of CycT1 could however also facilitate release of Brd4 from the chromatin and histone structures by offering an alternative binding site for the two Brd4 bromodomains. Such transition could liberate the P-TEFb–Brd4 complex from histones and possibly stabilize the elongation process by allowing P-TEFb to travel with the RNA polymerase. Moreover, lysine acetylation in the distal part of the CTD was recently described (55). These modifications could potentially act as binding sites for Brd4 bromodomains, recruiting thus the P-TEFb activator to RNAPII. Release of P-TEFb from the 7SK snRNP by a Brd4 construct comprising the C-terminal 154 residues has been shown before (10). This function requires the C-terminal helical element within the PID, as deletion of residues 1329–1345 abolished the activating function of human Brd4 (10). The deletion site harbors both the polybasic region of the PID as well as the leucine motif identified here and is thus in agreement with the results of the alanine mutagenesis experiments performed in this study.

Brd4 was recently shown to become phosphorylated at a conserved central region juxtaposed to the second bromodomain by casein kinase II (56). This modification was associated with a phosphor-switch trigger mechanism to unmask bromodomains and activator recruitment regions. The combined interaction of N- and C-terminal regions in Brd4 could in such a way also be modulated for P-TEFb binding and activation. The sequence similarity between Brd4 PID and HIV/SIV Tat yet raises the question if any RNA or DNA could sustain the PID-mediated P-TEFb activation effect, similarly as TAR RNA enforces the P-TEFb–Tat interaction. The arginine-rich motif of seven basic residues in Brd4 interspaced by a three-residue linker could interact with the phosphate groups of double-stranded oligonucleotides, similarly as Tat was found to bind to the major groove of TAR (43). The polybasic motif however could also interact with the acidic phosphorylation groups of the CTD and thus stimulate substrate recognition.

How Brd4 PID mechanistically achieves the stimulation of P-TEFb for CTD phosphorylation remains elusive at this point. The catalytic activity could be increased by enforcing the interaction with the substrate, e.g. by electrostatic interactions that would result in a higher affinity for the negatively charged CTD. A crystal structure of the N-terminal activation domain of AFF4 that recruits P-TEFb into the super elongation complex bound to P-TEFb showed binding to the CycT1 subunit but not to Cdk9 (57). The interaction is supportive of HIV-1 Tat binding to CycT1, whereas the *trans*-activator Tat was found before to displace the Cyclin TBD of Hexim1 by a mutually exclusive binding mechanism (45,46). These interaction schemes suggest that the N-terminal domain of AFF4 does not compete with binding to the proposed Hexim1 TBD binding site on CycT1 (58), and similar results were reported for the AFF1–CycT1 interaction (59). Similar to the two-pronged binding mechanism of Brd4, the cellular inhibitor Hexim1 interacts with both subunits of P-TEFb (11,40). While the C-terminal dimeric TBD of Hexim1 is necessarily required for recognizing CycT1, a highly conserved N-terminal PYNT motif inhibits the kinase activity of Cdk9 (40,^60^). The Brd4 PID might therefore solely interact with the active center of the kinase to achieve activation of P-TEFb in the presence of Hexim1. With the biochemical characteristics of the interaction domain determined, future studies will be designed to enlighten the structural details of Brd4 PID-mediated P-TEFb activation.

## SUPPLEMENTARY DATA

Supplementary Data are available at NAR Online.

Supplementary Data
