# Supplementary material for: Brd4 activates P-TEFb for RNA polymerase II CTD phosphorylation
Source: Nucleic Acids Res. 2014 May 23;42(12):7577–90. doi: 10.1093/nar/gku449 (PMC4081074; doi:10.1093/nar/gku449)
Supplement: Supplementary Data [file supp_42_12_7577__index.html]

Brd4 activates P-TEFb for RNA polymerase II CTD phosphorylation — Supplementary Data 

# Brd4 activates P-TEFb for RNA polymerase II CTD phosphorylation

## Supplementary Data

**Files in this Data Supplement:**

- Supplementary Data
