# Supplementary material for: Brd4 activates P-TEFb for RNA polymerase II CTD phosphorylation
Source: Nucleic Acids Res. 2014 May 23;42(12):7577–90. doi: 10.1093/nar/gku449 (PMC4081074; doi:10.1093/nar/gku449)
Supplement: Supplementary Data [file supp_gku449_nar-00649-x-2014-File010.pdf]

Supplementary Material to

Brd4 activates P-TEFb for RNA polymerase II CTD phosphorylation

Friederike Itzen[1], Ann Katrin Greifenberg[1,2], Christian A. Bösken[1,2] and Matthias Geyer[1,2,*]

[1]Max Planck Institute of Molecular Physiology, Department of Physical Biochemistry, 44227 Dortmund, Germany, [2]Center of Advanced European Studies and Research (caesar), Group Physical Biochemistry, 53175 Bonn, Germany.

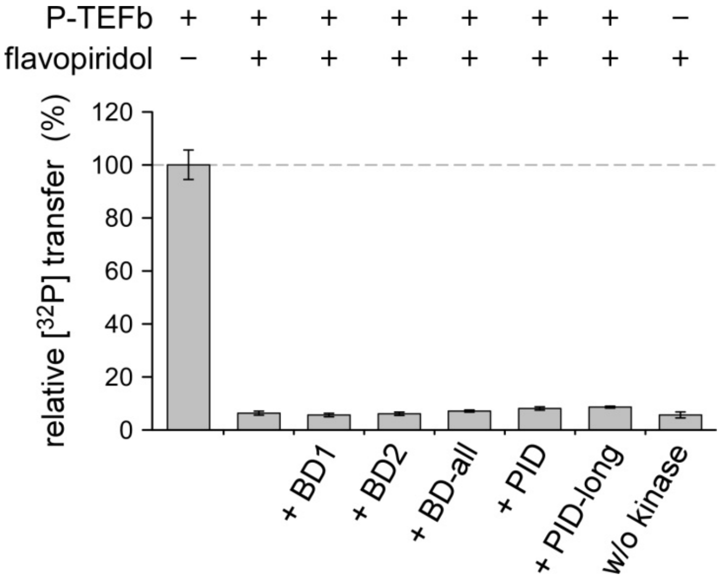

Supplementary Figure S1: Brd4 PID is unable to activate P-TEFb that has been inhibited by flavopiridol.

A kinase activity assay of P-TEFb for RNA polymerase II CTD phosphorylation was performed using radio-active labelled ATP. Flavopirodol potently inhibited P-TEFb as observed before. In contrast to the inhibiton of P-TEFb by Hexim1, addition of various protein constructs of Brd4 did not lead to kinase activation of P-TEFb in the presence of flavopiridol. This observation suggests a different mechanism of inhibition for the cellular protein Hexim1 compared to the ATP competitive compound flavopiridol.

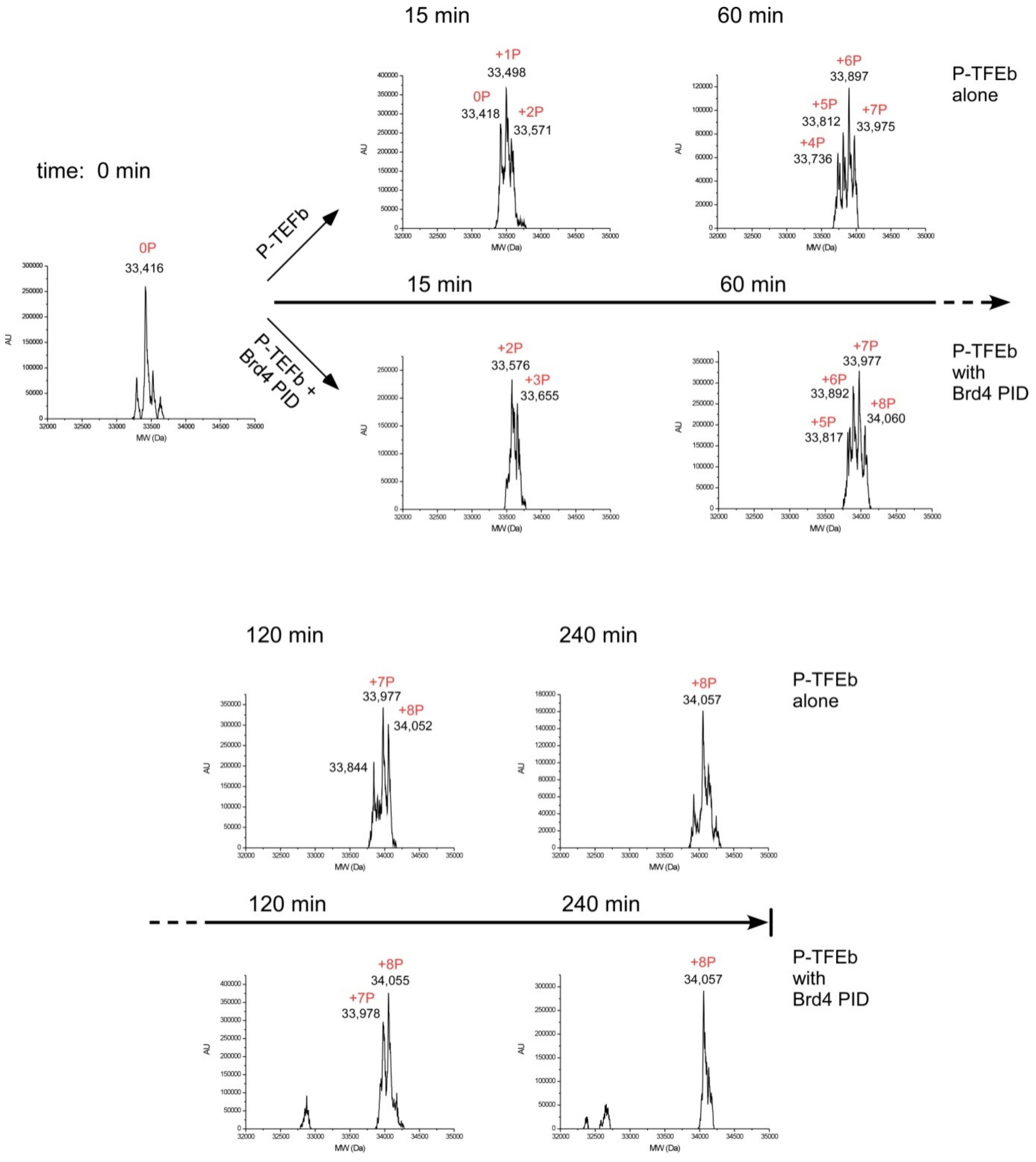

Supplementary Figure S2: Time course of the P-TEFb phosphorylation reaction in the presence or absence of Brd4 PID

ESI-MS monitoring of the increase in P-TEFb mediated CTD phosphorylation with and without Brd4 PID. A CTD substrate of eight full hepta-repeats was used as template. The kinase P-TEFb was applied at 0.2 µM concentration. The presence of Brd4 PID led to higher phosphorylation numbers at equal time points indicating the increased catalytic activity for RNAPII CTD. Upon reaction saturation both substrates exhibited the same number of phosphorylations, corresponding to one phosphorylation per hepta-repeat.

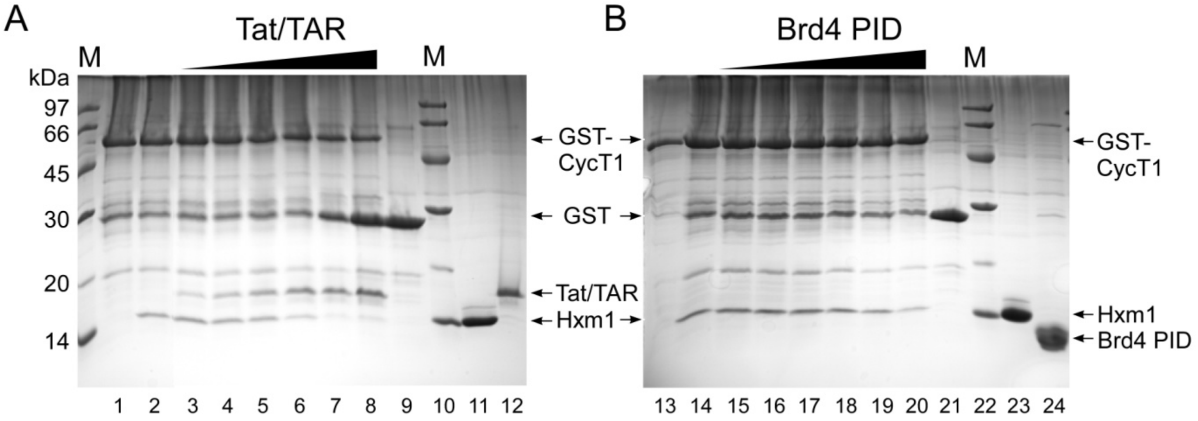

Supplementary Figure S3: The activator domain Brd4 PID is unable to displace the inhibitory Hexim1 protein from CycT1 binding.

SDS PAGE analysis of a binding competition experiment between Hexim1 (Hxm1) and either Tat/TAR (**A**) or Brd4 PID (**B**) for binding to GST-CycT1. Whereas the HIV-1 Tat/TAR complex is able to displace the Cyclin T-binding domain (TBD) of Hexim1 from CycT1 by binding to the same surface of the P-TEFb subunit, Brd4 PID does not co-precipitate with GST-CycT1 and is unable to displace Hexim1 from its binding site on CycT1. Increasing concentrations of either Tat/TAR or Brd4 PID were added in GST pull down experiments to the same amount of a preformed GST-CycT1–Hexim1 TBD complex. Bound proteins were co-precipitated with GSH beads and washed three times. Gels were stained with Coomassie blue.

A
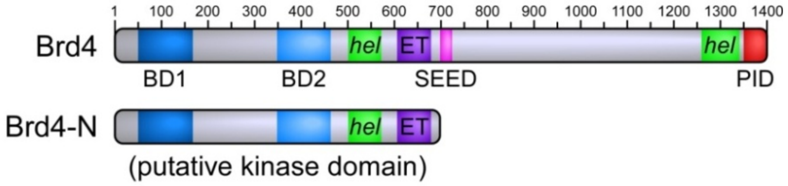

B
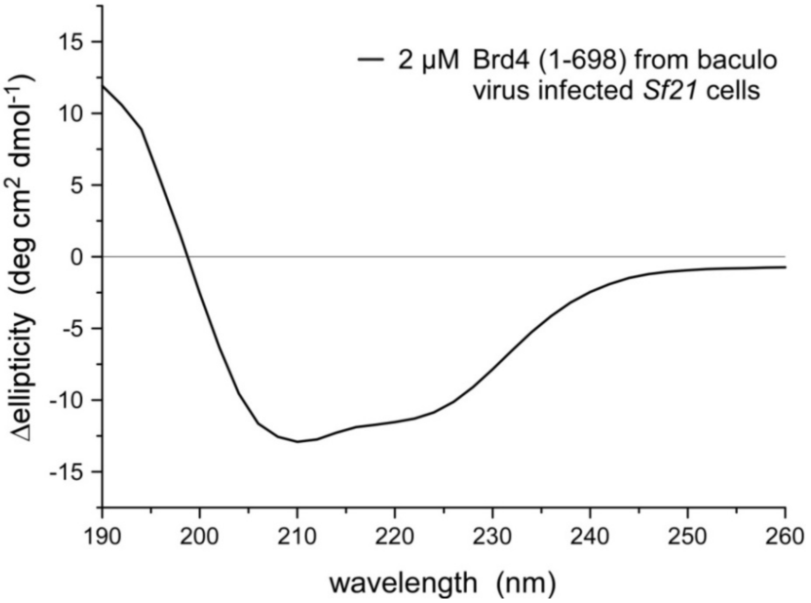

Supplementary Figure S4: Brd4 kinase domains and CD spectra of human Brd4 N-terminal domains.

(**A**) Cartoon of the putative atypical kinases domain 1-698 in human Brd4. Proteins were expressed in *E. coli* and baculo virus infected *Sf21* insect cells and purified to homogeneity. Protein products were confirmed by peptide mass fingerprint analysis. (**B**) CD spectroscopy measurement of the Brd4 1-698 protein expressed from *Sf21* insect cells. The ellipticity observed at a wavelength of 208 and 222 nm indicates a predominantly α-helically folded protein product.

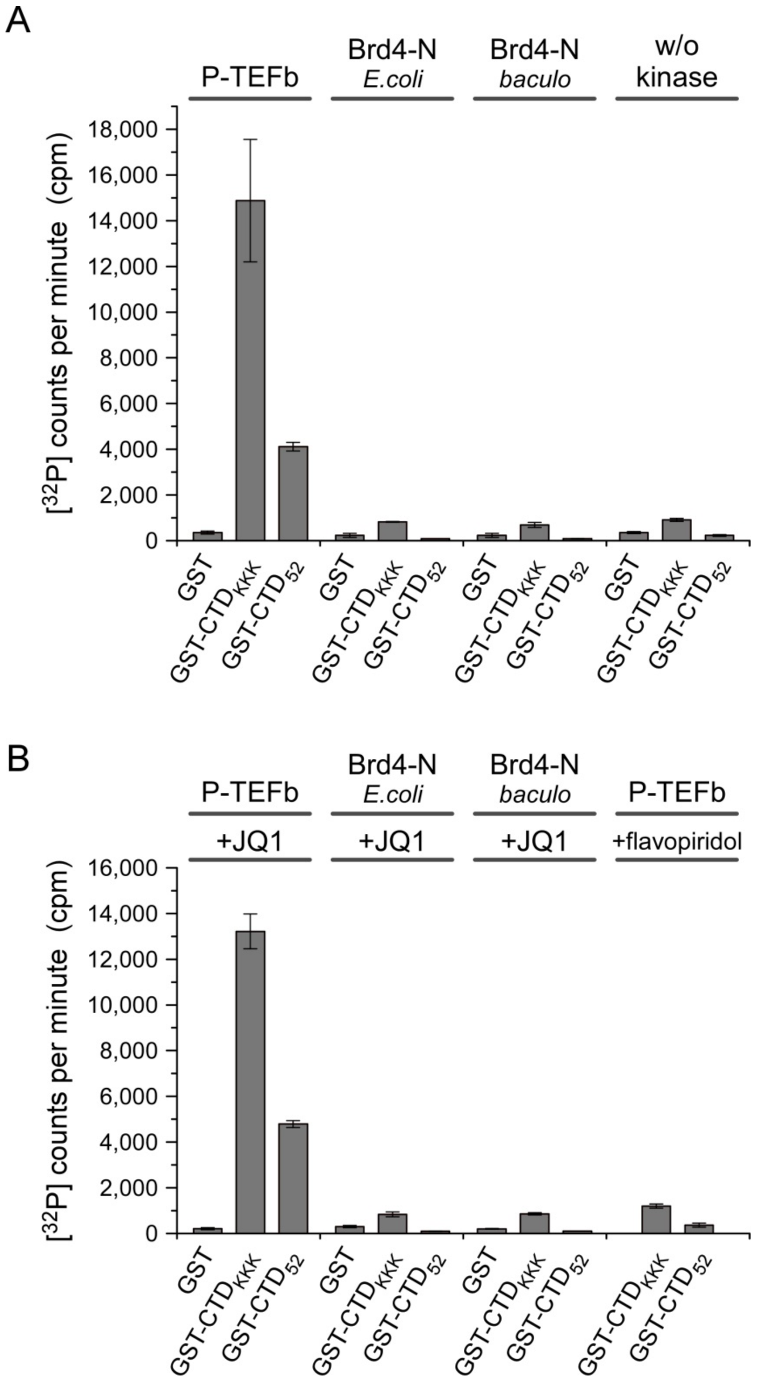

Supplementary Figure S5: Comparison of P-TEFb and Brd4 kinase activities for RNAPII CTD phosphorylation.

(**A**) P-TEFb and the putative kinase domain of human Brd4 from *E.coli* and baculo virus infected insect cell expression were incubated with either GST as control, a GST-CTD_{KKK} substrate that contained 8 hepta-repeats flanked by a poly-lysine stretch for improved filter binding capabilities, and full length GST-CTD_{52} with all 52 hepta-repeats from human. Whereas P-TEFb phosphorylated the CTD as observed before, the human Brd4 proteins showed no increase in CTD phosphorylation that exceeds the control measurements without kinase. (**B**) Addition of the bromodomain inhibitor JQ1 did not change the phosphorylation patterns, whereas flavopiridol potently inhibited the catalytic activity of P-TEFb as expected. Of note, the GST-CTD_{KKK} substrate with 8 hepta-repeats was supplied at 100 µM concentration whereas full length GST-CTD containing all 52 hepta-repeats was used at 10 µM concentration.
